# Novel Peptide Sequence (“IQ-tag”) with High Affinity for NIR Fluorochromes Allows Protein and Cell Specific Labeling for In Vivo Imaging

**DOI:** 10.1371/journal.pone.0000665

**Published:** 2007-07-25

**Authors:** Kimberly A. Kelly, Jonathan Carson, Jason R. McCarthy, Ralph Weissleder

**Affiliations:** Center for Molecular Imaging Research, Massachusetts General Hospital, Harvard Medical School, Boston, Massachusetts, United States of America; Center for Genomic Regulation, Spain

## Abstract

**Background:**

Probes that allow site-specific protein labeling have become critical tools for visualizing biological processes.

**Methods:**

Here we used phage display to identify a novel peptide sequence with nanomolar affinity for near infrared (NIR) (benz)indolium fluorochromes. The developed peptide sequence (“IQ-tag”) allows detection of NIR dyes in a wide range of assays including ELISA, flow cytometry, high throughput screens, microscopy, and optical in vivo imaging.

**Significance:**

The described method is expected to have broad utility in numerous applications, namely site-specific protein imaging, target identification, cell tracking, and drug development.

## Introduction

A number of advances in reporter and imaging technologies have led to the widespread use of fluorescent proteins (FP) and site-specific binders. Fusions of fluorescent proteins (e.g. green (GFP) or red fluorescent proteins (RFP)) and target proteins of interest have been used to study diverse cellular processes such as mitosis, DNA repair, cytoskeletal remodeling, receptor trafficking, focal adhesion, local calcium concentrations, membrane potential, pH, and microbial pathogenesis[1]–[3]. While such fusions demonstrate precise one-to-one targeting, they are constrained by the stability, fluorescence, and sensor properties of the mutant GFP, thus, their broader applicability as nanoscale indicators have some practical limitations. In addition, steric hindrance of FP in proximity to its target can potentially disrupt the native interactions of nearby proteins in multimeric complexes or alter cellular localization[4], [5].

An alternative to fluorescent fusion proteins is the use of site-specific small molecule labeling strategies. Recently developed methods have employed specific peptide handles to recruit small molecule ligands[6], [7], harnessed enzyme activity to catalyze conjugation of tags[8], [9], or made use of cellular protein machinery[10], [11]. Some of these methods are particularly useful for in vivo imaging in whole organisms[8]. Higher affinity, increased target-to-background ratios and one-step reactions that do not rely on exogenous enzymes[12], [13] would further enhance the utility of these approaches.

Organic fluorochromes are intriguing molecular probes, as they do not suffer from many of the detrimental aspects of FP. These dyes have played an essential role in immunofluorescence, analytical testing, and other emerging imaging applications. In particular, far red (FR) and near infrared (NIR) fluorochromes containing (benz)indolium subunits have proven useful in a multitude of *in vivo* imaging applications, as light penetrates tissue more efficiently and because tissue autofluorescence is much lower in this range[14], [15]. To expand the utility of these fluorochromes, it would be useful to develop biological ligands tailored specifically to the structure of the dye, ideally with affinity constants surpassing those of monoclonal antibodies. Thus, the discovery of short peptides specific to organic fluorochromes through library screening may a) provide a minimally invasive way of “tagging” proteins, b) enable imaging of intracellular proteins and c) provide specificity since endogenous proteins should not recognize NIR dyes.

**Figure 1:**
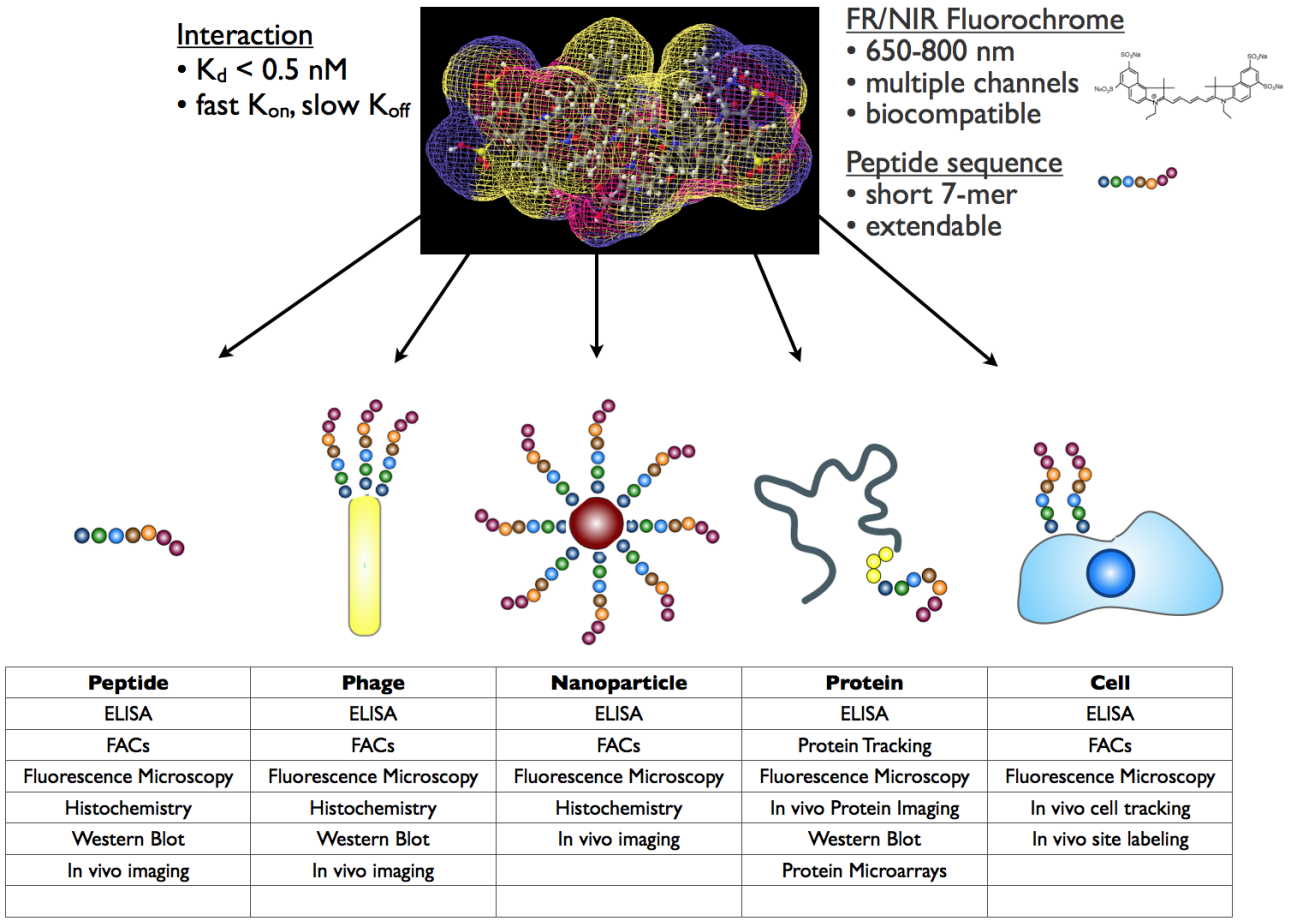
Bioapplications of the IQ-tag-benzindolium fluorochrome binding pair.

In the current study we use phage display screening to determine whether peptides with sufficiently high affinity for (benz)indolium-derived fluorochromes could be identified. We found that screening a 7-mer library results in convergence onto a single peptide sequence with subnanomolar affinity for these dyes. Furthermore, the utility of the peptide for analytical testing, cell labeling, and in vivo imaging is demonstrated. The developed peptide sequence should be broadly applicable in a variety of analytical and imaging applications (Fig. 1).

## Results

**Figure 2:**
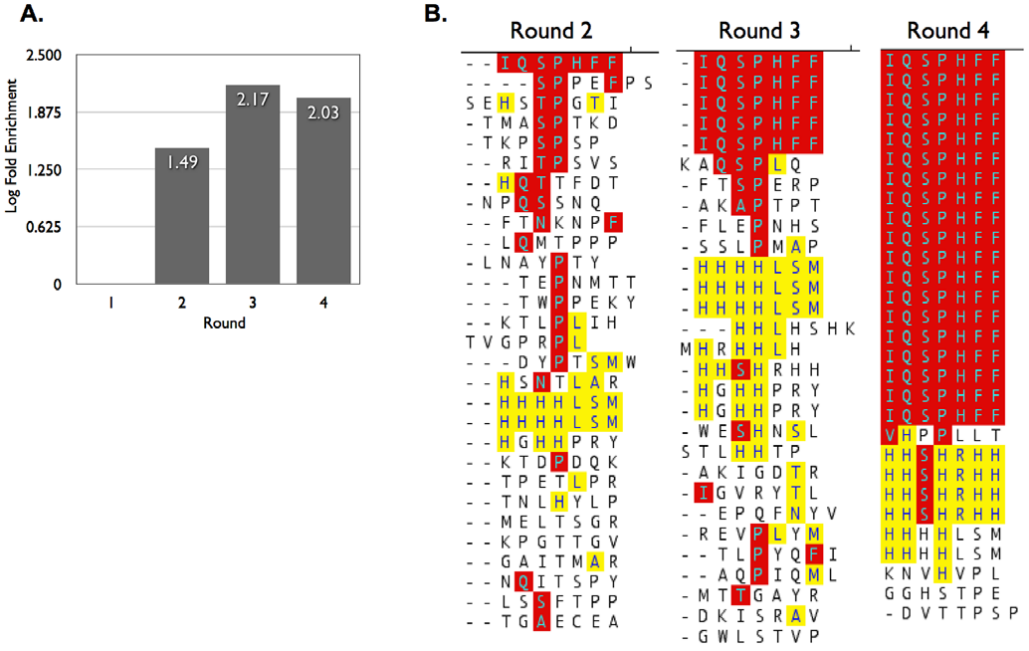
Phage display selection and evolution of IQ-tag phage. A. Fold enrichment of NIR fluorochrome (BSA-GH680)-selected phage. B. Sequences of randomly selected phage clones isolated from biopanning rounds 2, 3, and 4. Note appearance of two families containing (Q)SP/TP and HXHH, and expansion of clone IQSPHFF.

Utilizing a linear, random 7 amino acid phage display library to first select against a model (benz)indolium fluorochrome (GH680), we observed >100-fold enrichment in the third and fourth rounds of panning, suggestive of a successful selection (Fig. 2A). Moreover, identification and alignment of amino acid sequences of individual clones from rounds 2, 3, and 4 revealed a robust enrichment and narrowing to a small area of the peptide diversity space. Alignment of the sequences from round 2 showed lack of a single consensus family, however by the third and fourth round, one had emerged (Fig. 2B). In the fourth round, 63% (19/30) of the peptides contained the sequence IQSPHFF (IQ-tag). Although round two did not have a clear consensus, the IQSPHFF sequence was indeed present. In addition to the IQSPHFF peptide, a second although less prevalent (20%; 6/30) motif consisting of HHS/HHXH was also identified through the selection (Fig. 2B).

**Figure 3:**
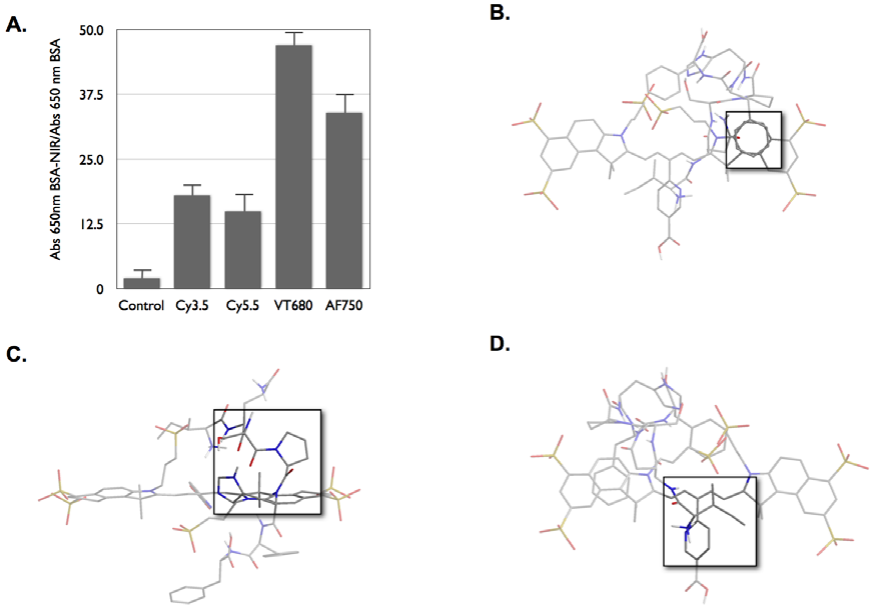
Molecular modeling of IQ-tag binding to benzindolium fluorochrome. A. Binding of IQSPHFF phage to BSA-conjugated fluorochromes via ELISA. B. π-stacking between the phenylalanines of the peptide and the benzidolium subunit of GH680 C. SPH amino acids extend over the linker of GH680 to the opposite face of the fluorochrome D. Interaction of the hydrophobic isoleucine residue and the polymethine linker of the fluorochrome.

Once identified, the affinity of the peptide to a variety of (benz)indolium fluorochromes (Cy3.5, Cy5.5, GH680, and Alexa Fluor 750 (AF750)) was determined. Fig. 3A illustrates the binding affinity of each dye for the IQ peptide on phage, with GH680 exhibiting the highest affinity. This binding affinity was trailed closely by AF750, whereas Cy3.5 and Cy5.5 both exhibited approximately four-fold less binding than GH680. To better understand the differences in affinity, we performed molecular modeling, assuming that the fluorochromes were relatively rigid active sites to which the peptide “ligand” was bound. In comparing the resulting structures, several putative binding interactions became apparent (Fig. 3B–D). The C-terminus of the peptide, containing the hydrophobic and aromatic phenylalanine residue, interacted with one of the (benz)indolium subunits via π-π interactions (Fig. 3B). The next three amino acids, SPH, extended over the alkene linker of the cyanine dyes to the opposite face of the fluorochrome, with the hydrophilic portions directed away from the core (Fig. 3C). Lastly, the hydrophobic N-terminal isoleucine interacted with the similarly hydrophobic alkene linker (Fig. 3D). In the case of Cy 3.5, this last interaction was not observed, which is most likely due to the length of the linker and steric strain (the length of the linker in Cy 3.5 is 3 carbons, in Cy 5.5 and GH680 is 5 carbons, and in AF750 is 7 carbons; Fig. S1). Similar to Cy 3.5, the interaction between the peptide and Cy 5.5 is lessened by a sub-optimal interaction of the isoleucine with the flexible hexanoic acid moiety used in the conjugation of the fluorochrome (Fig. S1B). For GH680 (Fig. 3B–D) all three interactions were viewed as optimal.

**Figure 4:**
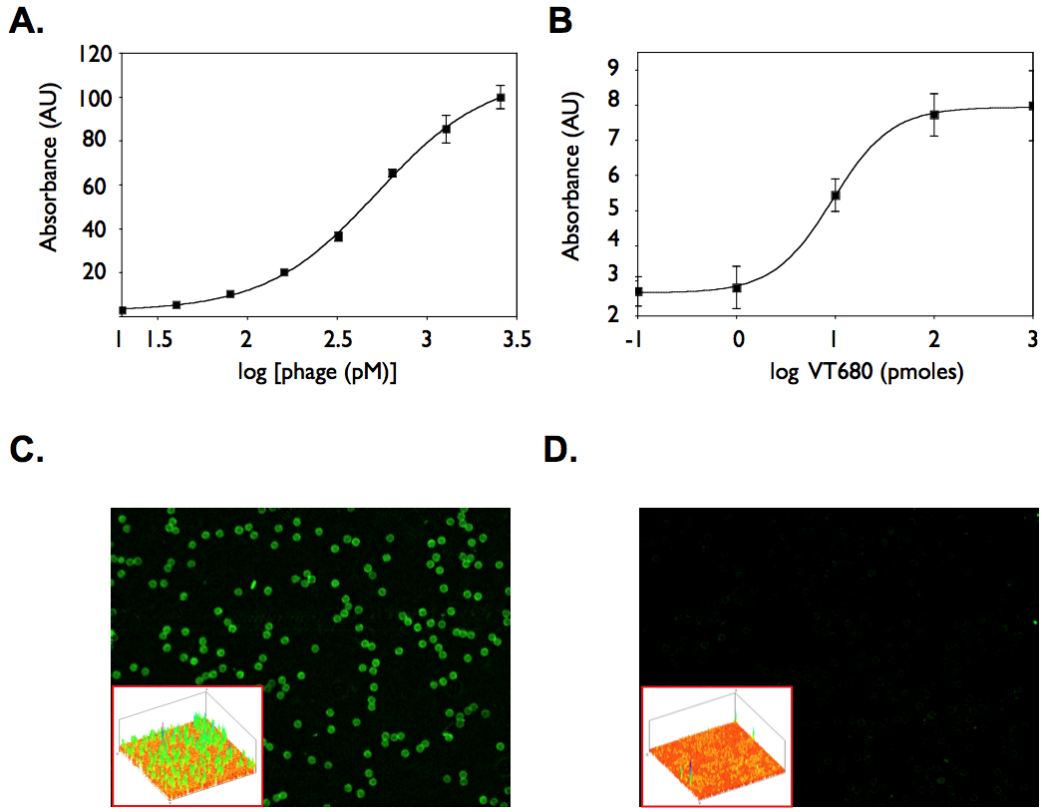
IQ-tag binds with high affinity to GH680 and permits imaging via fluorescence microscopy and FACs. A. Representative sigmoidal dose response curve of IQ-tag binding to the benzindolium fluorochrome (K_D_ = 0.53±0.12 *SD*). *Bars, SEM*. B. Immunodetection of GH680 on a glass slide via IQ-tag phage and anti-M13. Integrated pixel density of TMB substrate deposition via anti-M13-HRP reactions on spot dilutions of GH680. C. beads conjugated to GH680 or D beads conjugated to BSA were incubated with IQ-tag displaying FITC labeled phage and imaged via fluorescence microscopy (20x objective, equal exposure, FITC channel). Insets: Pixel topography renditions of the images from C and D. Average fold FITC intensity comparing dye-conjugated and unconjugated beads is 6.1 (p<10^−36^, *t*-test).

The dependence on context of IQ-tag binding to GH680 was characterized next. First, we determined the affinity of IQ-tag displayed on phage to immobilized GH680-BSA. The binding curves formed a classic sigmoidal shape indicative of specific binding of the dye to IQ-tag with an average K_D_ of 0.53±0.2 nmol/L. To elucidate whether this binding was specifically due to the presentation of IQ-tag on the phage (Fig. 4A), we cloned the sequence encoding this peptide on the p7 coat proteins located on the opposite end of the phage. Phage expressing N′-p7-AIQSPHFF likewise demonstrated a sigmoidal dose response (r^2^ = 0.984) with high affinity (K_d_ 0.53±0.12 nmol/L). In contrast, phage without displayed peptides demonstrated negligible binding. To estimate the stoichiometry of the dye binding to phage displaying IQ-tag, a known concentration of phage was incubated with the dye. This resulted in approximately one fluorochrome per peptide (7.4±2.0, *SD*).

To further investigate the molecular interactions of IQ-tag with NIR fluorochromes, we applied dilutions of GH680 to chemically coated glass slides and used them in colorimetric immunoassays (Fig. 4B). The (benz)indolium dye spotted glass slides were incubated with IQ-tag displaying phage and the binding was detected with anti-M13-HRP. Under these conditions, concentration dependent binding of the targeted phage to GH680 was observed. In this application, the peptide had a half-maximal response of 10 pmol and a detection limit of 5 pmol of fluorochrome (Fig. 4B). GH680 was next immobilized on polystyrene microbeads and was interrogated with fluorescein (FITC) labeled IQ-tag displaying phage. Fluorescence microscopy showed strong binding of phage to the dye-modified beads (Fig. 4C), but not to unmodified control beads (Figure 4D) (p<10^−36^, *t*-test). These data further suggest that the IQ-tag motif specifically binds to (benz)indolium dye, itself, and is not dependent on the presence of BSA.

**Figure 5:**
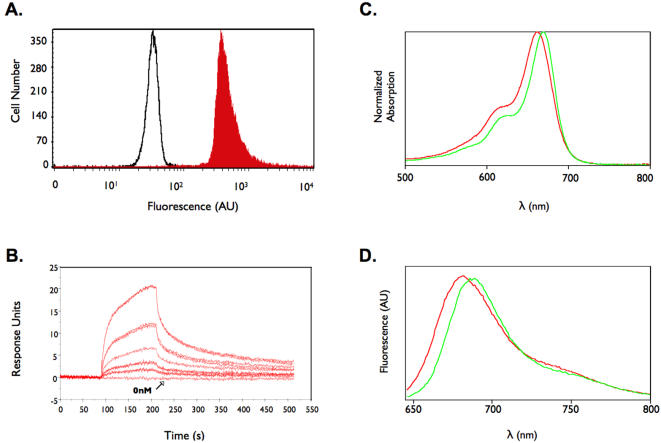
Characterization of IQ-tag peptide/GH680 interaction. A. IQ-tag-Biotin peptide was incubated with NIR fluorochrome-conjugated Bang's beads (red histogram) or BSA conjugated Bang's beads (black histogram), then incubated with streptavidin-FITC and analyzed via flow cytometry. B. BiaCore experiments. IQ-tag-biotin was immobilized on a streptavidin chip then decreasing concentrations of GH680 were flowed over the surface (640 nM to 40 nM, bottom line buffer only control). C and D. Photochemical properties of GH680 (green trace) and GH680+IQ-tag (red trace). GH-680 binding to IQ-tag blue shifts the absorption by 14 nm.

The high affinity observed in phage is not always correlated with the same affinity in the monomeric and free form of the peptide due to avidity effects as a result of multiple copies of the peptide on the phage surface. Therefore, we synthesized soluble, free monomeric peptide corresponding to the sequence IQSPHFFGGSK(biotin) (IQ-tag) and asked whether the peptide could probe GH680 modified polystyrene beads. Flow cytometry results depicted in Figure 5A demonstrate that this is indeed possible. To further corroborate this result and to demonstrate the strength of the interaction, we asked whether IQ-tag -NIR complexes could form and co-elute utilizing C18 reverse phase HPLC. Free NIRF eluted immediately after the void volume of the column. In contrast, when peptide and NIRF were incubated then injected, the peak corresponding to fluorochrome shifted and was retained longer, indicating the binding of fluorochrome to peptide (Figure S2). Surface plasmon resonance analysis showed a saturable, dose-dependent response with an affinity of the monomeric peptide binding to GH680 of approximately ∼100 nmol/L (Fig 5B). We next examined the NIRF fluorescence shift and relative quantum yield upon binding (Fig 5C and 5D). Binding of IQ-tag to GH680 blue shifted the absorption by 14 nm and increased the relative quantum yield from 0.89 to 1, a 12% increase.

**Figure 6:**
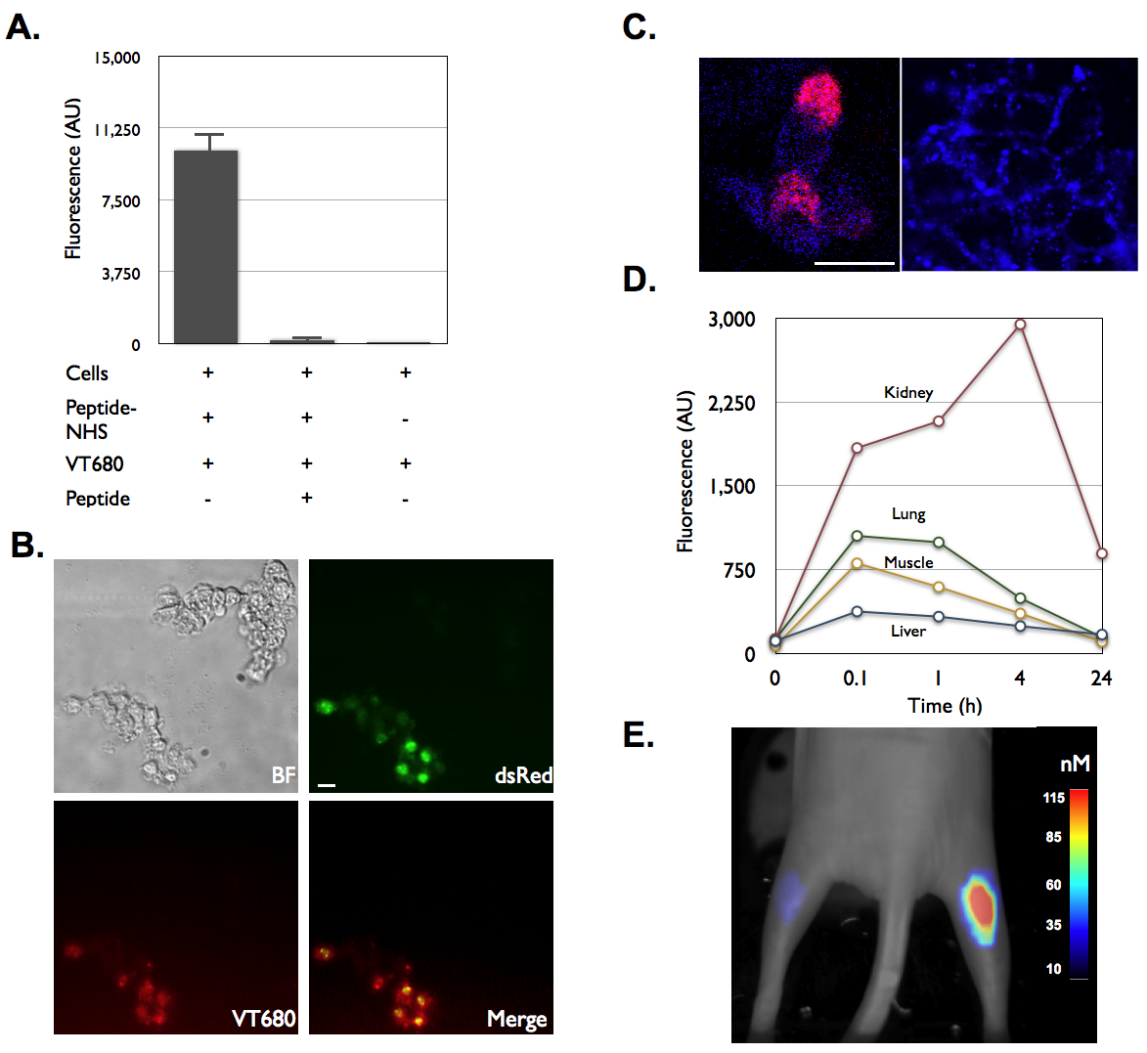
Peptide-NIR dye pair allows for the in vitro and in vivo detection of cells. A. Vehicle treated and IQ-tag modified HT1080 cells were incubated with GH680. The presence of the (benz)indolium fluorochrome was detected via a fluorescence plate reader. B. HEK-293T cells with the expression of dsRed and IQ-tag peptide fused to PDGFR were imaged via fluorescence microscopy for their ability to bind GH680. C. Confocal microscopy showing membrane localization of GH680. Scale bars: 10 µm D. Non-IQ-tag animals were injected tail vein with GH680 and organs removed at indicated time points post injection to quantify fluorochrome concentrations. E. Mice implanted with HEK-293T cells expressing IQ-tag peptide fused to PDGFR (right flank) or without expression (left flank) were injected with GH680 then imaged via FMT.

To determine whether IQ-tag may be useful for imaging cells, we first conjugated the N-hydroxysuccinimide ester functionalized peptide sequence (IQ-tag-NHS) onto HT1080 colon adenocarcinoma cells and incubated cells with 1 nmol of GH680 (Fig. 6A). Peptide labeled cells bound fluorochrome greater than 120 fold better than unlabeled cells. In addition, free unconjugated peptide was able to compete for 98% of the bound dye (Fig. 6A). A bicistronic construct expressing dsRed and a fusion protein with IQ-tag fused to the amino terminus of the platelet-derived growth factor receptor (PDGFR) transmembrane domain was subsequently created to directly image protein expression and tumor cells. Transfection and expression of the peptide was confirmed by the co-expression of dsRed. HEK-293T cells that were expressing dsRed, also bound benzindolium fluorochrome (Fig. 6B). GH680 did not bind to cells devoid of dsRed expression. In addition, cells were found to bind GH680 in the plasma membrane, as visualized by confocal microscopy (Fig. 6C). This construct was further extended for in vivo visualization and tracking of cells and the PDGFR protein. Biodistribution experiments show that GH680 was rapidly eliminated via renal excretion with lung, muscle, and liver fluorescence minimal within 4 hours after injection (Fig. 6D). Importantly there was no non-specific binding to muscle tissue where titin (a protein with the nearest sequence to IQ-tag) is expressed abundantly. To determine whether we could image cells in vivo using this system, IQ-tag expressing cells were first implanted subcutaneously into nude mice, followed by a systemic injection of GH680 and imaging by fluorescence mediated tomography (FMT) (Fig. 6E). After intravenously injecting GH680, IQ-tag expressing cells became brightly fluorescent with the signal persisting for over 24 hours whereas contralaterally implanted control cells showed negligible fluorescence (42.2 nM vs 1 nM in control leg; p<0.0001).

## Discussion

A number of reporter and imaging technologies have recently been developed to visualize site-specific proteins and cellular trafficking (reviewed in[2], [13]). Specific examples include protein tags, such as tetracysteine or hexahistidine motifs, which are recognized by bi-arsenic-derivitized fluorescein[16], his-tags for Ni-NTA-conjugated fluorochromes[17], or various forms of biotin ligases that are revealed with labeled avidins[3], [8]. Collectively, these methods have allowed unprecedented insight into cellular protein trafficking. Based on the hypothesis that it is feasible to develop affinity ligands to commercially available (benz)indolium fluorochromes that are commonly utilized in vivo, we performed de novo phage screens for high affinity binding peptides. Here we report on a novel peptide tag for NIR dyes, which is short, linear, and does not occur in nature.

The identified IQ-tag peptide (IQSPHFF) is unique and has a sub-nanomolar binding affinity for the NIR fluorochrome GH680, due to a number of optimal binding interactions between the peptide and benzindolium dye. According to results obtained from molecular modeling studies of the docked ligand, π-stacking between the phenylalanine residues of IQ-tag and the (benz)indolium subunit of the fluorophore yields preferential orientation of the peptide on the dye. The next three amino acids, SPH, extend over the alkene linker of the dye to the opposite face with the hydrophilic portions directed away from the core. Finally, the hydrophobic N-terminal isoleucine is able to interact with the similarly hydrophobic polymethine linker. When the binding of the peptide to various other cyanine dyes (AF750, Cy5.5, Cy3.5) is investigated, varied affinity constants are obtained. These differences in affinity, as well as alterations to the peptide sequence, could be used to identify IQ-tag like sequences with even higher affinity or subsets with affinity for specific NIR fluorochromes. For example, the use of unnatural amino acids or additional screens with mRNA display[18] could be used to yield additionally evolved compounds. The somewhat hydrophobic IQ-tag can also be modified at either its N- or C-terminus in order to modulate the polarity of the peptide.

In order to determine whether the IQ-tag peptide has homology to any sequence naturally occurring in the mammalian genome, a Basic Local Alignment Search Tool (BLAST) search of the human and mouse proteomes was conducted. Five out of the seven amino acids were found to match a sequence within titin, a giant polypeptide found in striated muscle cells. Molecular modeling of the titin peptide on GH680 failed to demonstrate the same binding mode as the IQ-tag peptide, intimating the importance of the serine and two terminal phenylalanine residues. Therefore, it is unlikely that this sequence will exhibit as exquisite an affinity for benzindolium fluorochromes as the 7-mer IQ-tag. Thus, few restrictions may exist for biological applications of the IQ-tag/NIR fluorochrome interaction.

The dye sensing ability of the IQ-tag system is amenable to a number of different modalities (Fig. 1). For example, we envision the use of cell permeable fluorochromes for detection of the soluble peptide fused to cytoplasmic proteins, expression of the peptide on prokaryotic cells, multivalent display of IQ-tag on a variety of nanomaterials and sensors, site-specific protein expression, and eukaryotic cell tagging applications. Furthermore, the IQ-tag sequence, modified with a non-cyanine dye-based fluorochrome, radioisotope, or biotin, could also be used as an amplification strategy to determine (benz)indolium fluorochrome localization and/or concentration. Another possibility, as shown in our cell experiments, is the inclusion of this sequence as a handle on an engineered protein to permit in vitro or in vivo protein or cellular imaging. If the tagged protein is an internalizable receptor, growth factor, viral coat protein, or peptide toxin, an avenue for detailed and live imaging of intracellular receptor or drug trafficking becomes plausible. The described IQ-tag technology is anticipated to be particularly useful for in vivo experimentation, as the transparency of animal tissue increases at far-red and near-infrared wavelengths. In total, IQ-tag represents a system, which is expected to have immediate, broad biological application.

## Materials and Methods

### Materials

Cy3.5 and Cy5.5 were obtained from GE Healthcare (Niskayuna, NY). Alexa Fluor 750 was obtained from Invitrogen (Carlsbad, CA). Genhance 680 (GH680) was obtained from VisEn Medical (Woburn, MA). Polystyrene particles were obtained from Bang's Laboratories, Inc (Fishers, IN). Amino functionalized slides were from Sigma. All molecular biology and cloning reagents were from New England Biolabs (Beverly, MA), except Omnimax T2 Ultracompetent Bacteria (for transformation and RFDNA preparations) and custom oligonucleotide primers, which were from Invitrogen. Rapid DNA Ligation Kits, which were obtained from Roche (Indianapolis, IN). All other chemicals were from Fisher Scientific or Sigma-Aldrich.

### Phage selection

We used a phage library that expresses random 7-amino acid sequences on the N-termini of all 5 p3 coat proteins. For selection, we covalently coupled GH680 to BSA and immobilized the conjugate on Nunc Maxisorp plates (Fisher). In addition, we prepared a subtraction well containing a BSA-only sample. After a subtraction step designed to filter BSA-and/or plastic-binding clones from subsequent pans, four successive rounds of binding, elution, and amplification were performed.

### Phage ELISA

NHS ester of GH680, Cy3.5, Cy5.5 or AF750 (1 mg) was conjugated to BSA (100 mg/mL BSA, 0.3M NaCO_3_ buffer, pH 8.6, 1hour 37°C) under reaction conditions of approximately tenfold molar excess fluorochrome to BSA. BSA-fluorochrome conjugate or BSA was adsorbed (5.0 µg/0.1 ml per well) overnight to wells of a Nunc Maxisorp plate (Nunc, Rochester, NY). Supernatants were discarded and blocked (5% BSA, 0.3M NaCO_3_, pH 8.6, RT, 45 min). After two washes (PBS), 1:2 serial dilutions of phage (∼10^11^ to ∼10^9^ PFU/well) in DPBS+1% BSA were added to wells and incubated for 1 hour. Subsequent to incubation, wells were washed (5×PBS+0.05% Tween-20) and incubated (1:5,000, DPBS+1% BSA) with anti-M13 conjugated to HRP (GE Biosciences, Piscataway, NJ) for 45 min. Next, wells were washed again (5×PBS+0.05% Tween-20) and developed with tetramethylbenzidine (TMB), and absorbance at 650 nm was determined (Emax, Molecular Devices, Sunnyvale, CA). Mean difference between absorbances in the BSA-Fluorochrome containing wells and the BSA-only wells were calculated. EC_50_ values were obtained using sigmoidal dose response (variable slope) curve fitting from Prism 4 (GraphPad Software, San Diego, CA).

### Cloning

To generate IQSPHFF peptide fused to the N-terminus of g7 splice overlap extension PCR was performed using M13KE RFDNA according to manufacturer's instructions (New England Biolabs, Beverly, MA). p7-IQSPHFF-containing phage clones were positively screened by mobility shift in PCR and confirmed by DNA sequencing.

### Fluorescence Microscopy

Polystyrene beads conjugated to NHS-GH680 (NaHCO_3_ buffer, pH 8.6) or unconjugated control beads, were blocked with 5% BSA, washed, and incubated with FITC-labeled IQSPHFF phage. Following incubation, beads were washed 5x, and supernatants were discarded. Beads were then dissolved to approximately 5,000 per µL, plated in a 96-well plate, and viewed by fluorescence microscopy using the 20× objective (Axiovert 100 TV, Zeus, Thornwood, NY). To obtain quantifiable fluorescence data from microscopic images, randomly selected bead images were analyzed for integrated pixel density (IPD) via Image J software.

### Colorimetric Coated Slide Assay

Serial dilutions of GH680 were spotted on an amino functionalized glass slide (Sigma-Aldrich) and were incubated overnight (4 °C), allowed to air dry, and then blocked with 5% BSA (0.3M NaCO_3_, pH 8.6) for 1 hr, RT with gentle agitation. Following this, the slide was rinsed with DPBS and incubated with 10^12^ PFU of IQ-tag phage suspended in DPBS for 2-3 hrs. Supernatants were discarded and the slide was washed (3×5 min.) with DPBS plus 0.05% Tween-20. Next, anti-M13-HRP was added to the slide (1:5,000+5% BSA in DPBS+0.05% Tween-20) and mixed with gentle agitation (45 min.) Following 4 washes (DPBS+0.05% Tween-20; final wash dH_2_0), the slide was developed with TMB. Images of the slide were captured with a digital camera (Nikon CoolPix).

### Flow Cytometry

To verify binding of free biotinylated, IQ-tag peptide to GH680 via FACS, 10^6^ polystyrene Bangs beads per reaction were labeled with GH680 (10^10^ fluorochromes per bead, 0.1 nmol per mL in 160 mL 0.3 M NaCO_3_, pH 8.6 1h RT), washed (3x), blocked (5% BSA, 0.3M NaCO_3_, pH 8.6, 1 hr.), resuspended in binding buffer (100 mL DPBS+0.05% Tween 20+1% BSA) containing 1mg of IQSPHFF peptide (+/−), washed (3× 500 mL DPBS+0.05% Tween 20), probed with FITC-Streptavidin (1hr. at RT X 1:200 in DPBS+0.05% Tween 20+1% BSA), resuspended in DPBS, and analyzed via FACS.

### Molecular Modeling and Peptide Characterization

All molecular modeling, minimization, and docking were accomplished in Cache 6.1 (Fujitsu, Tokyo, Japan). The ground state geometry of the peptide (IQSPHFF) and each fluorochrome was calculated using CONFLEX/MM3 (medium search) parameters. The peptide was docked onto the fluorochrome using the dye as a rigid active site, while the peptide was identified as the flexible ligand. Once docked, the geometry was further optimized using the CAChe FastDock algorithm. All experiments were performed in triplicate to ensure consensus of the binding motif. For spectroscopic experiments, the fluorescence emission spectrum of GH680 or GH680/IQ Tag (ratio of 1:1) in distilled water was obtained and the integrated area under the curve (AUC) calculated. The relative fluorescence quantum yields were calculated from the ratio of the AUC of the two solutions. All measurements were performed in triplicate. For surface plasmon resonance experiments, biotinylated peptide was captured onto an avidin/dextran surface followed by injection (640 to 40 nM) then continuous flow of GH680 over both control and active surfaces (BiaCore, GE, Cambridge, MA).

### Synthesis of IQSPHFFGGSK peptide

IQSPHFFGGSK(biotin) IQ-tag peptide was purchased from Tufts Peptide Core Facility and used as received. To produce the N hydroxysuccinimide functionalized peptide, Ac-IQSPHFFGGSK(ivDde)G-Su was synthesized on an automated solid phase peptide synthesizer (433A, Applied Biosystem, Foster City, CA) employing the traditional Fmoc methodology on Fmoc-Gly-Wang Resin (154 mg, 0.1 mmol). Upon completion of the synthesis, the peptide-resin in DMF (4 mL) was acetylated using acetic anhydride (30 µL, 3.3mmol) and diisopropylethylamine (DIPEA, 40 µL, 3.8 mmol). The reaction proceeded for 30 min, at which point the resin was washed twice with DMF and reacted a second time using the conditions listed above. The resulting acetylated peptide resin was cleaved from the resin using 95% TFA/2.5% triisopropylsilane (TIS)/2.5% H_2_O for 2 h, filtered to remove the resin, and precipitated in methyl-*tert*-butyl ether (MTBE). The precipitate was dissolved in DMF (4 mL), and reacted with N-hydroxysuccinimide (NHS, 58 mg, 0.5 mmol), dicyclohexylcarbodiimide (DCC, 103 mg, 0.5 mmol), and dimethylaminopyridine (DMAP, 61 mg, 0.5 mmol) for 1 h. The solution was filtered through cotton wool to remove dicyclohexylurea, and precipitated with MTBE (10 mL) to yield the crude product. The crude product was then purified via HPLC then subjected to mass spectroscopic analysis to verify molecular weight. +ESI-MS (60 V, MeOH) m/z = 1606.8 (MH^+^).

### Cell Labeling

HT1080 cells (20,000 cells/well) were washed 3x with PBS to remove media then incubated with 0.1 mL of 100 µM of Ac-IQSPHFF-NHS peptide (IQ-tag-NHS) or vehicle (PBS with 1% DMSO) for 1 hour at 37°C. The NHS functional group will covalently conjugate the peptide onto proteins with free amine groups on the cells. Subsequent to incubation, cells were washed then incubated for 1 hour at 37°C in the dark with 1 nmole of GH680 or 1 nmole of GH680+5 nmoles of underivatized peptide (competition experiment). Cells were then washed 6x with PBS+0.1 % Tween 20 and analyzed via fluorescence plate reader GeminiXS (Molecular Devices).

Vector DNA containing bicistronic mammalian expression cassettes encoding ds-Red and a PDGFR transmembrane domain chimera that exposes the IQSPHFF peptide were transfected accordingly. For confocal microscopy imaging of HEK-293T cells, 100,000 cells per well of a 4-well chamber slide (Nunc, Rochester, NY) were plated then transfected 16hrs later using Lipofectamine2000 reagent at a concentration of 1.2 µg DNA and 2 µl of Lipofectamine per well, according to manufacturer's instructions. 68h after transfection, wells were washed gently with DPBS and replaced with 200 µl of DPBS containing 50 µmol/L GH680 680 and 1%BSA for 1h at 37C. Treated wells were then washed (3x DPBS+1% BSA+0.01% Tween-20), fixed (5–10 min.×DPBS+2 % paraformaldehyde), then imaged via the 20x objective of the Nikon Axiovert-100 inverted microscope and IPLab software and via the 40x objective of a Zeiss LSM Pascal confocal microscope (Carl Zeiss, Thornwood, NY).

### In Vivo Imaging

For in vivo imaging of HEK-293T cells, 5 million cells were transfected into twin wells of a 6-well plate with parameters scaled to those described above. IQ-tag-PDGFR transfected and mock transfected cells were injected locally for imaging and then probed via systemic injection of GH680 (2 nmoles). Imaging was performed by fluorescence mediated tomography (FMT). Mice were anesthetized by inhalation anesthesia (2% isoflurane, 1 L/min O_2_) using an isoflurane vaporizer (Braintree Scientific, Braintree, MA). FMT experiments were performed using a commercially available imaging system (VisEn Medical, Woburn, MA). Data sets were acquired at wavelengths 680/700 nm excitation/emission in anesthetized mice. Image data sets were reconstructed using a normalized Born forward model adapted to small mouse models[19]. Image acquisition time per animal was 2 minutes and reconstruction time was 1–2 minutes. Images were displayed as raw data sets (excitation, emission, masks) and as reconstructed 3D data sets in axial, sagital and coronal planes. Fluorochrome concentration in the target was automatically calculated from reconstructed images and expressed as pmol fluorochrome/defined target volume. Biodistribution experiments were conducted after systemic administration of GH680 (2 nmoles) by removing organ samples and measuring fluorochrome concentrations at different time points.

## Supporting Information

Figure S1 Molecular modeling of IQ-tag peptide binding to representative fluorochromes. A. sub-optimal IQSPHFF docking with Cy 3.5 (3-carbon linker too short). B. sub-optimal IQSPHFF docking with Cy 5.5 (isoleucine-hexanoic acid interference). C. IQSPHFF docking with AF750.
(0.21 MB TIF)

Figure S2 Analysis of IQSPHFF peptide binding to NIRF via HPLC. Free GH680 (upper trace) or GH680 incubated with IQ-tag (lower trace) were analyzed via HPLC with a reverse phase C18 column. Note the shift in retention time for NIRF incubated with peptide indicating the formation of a stable complex.
(0.11 MB TIF)
