# Supplementary figures and images for: Novel Peptide Sequence (“IQ-tag”) with High Affinity for NIR Fluorochromes Allows Protein and Cell Specific Labeling for In Vivo Imaging
Source: PLoS One. 2007 Jul 25;2(7):e665. doi: 10.1371/journal.pone.0000665 (PMC1919420; doi:10.1371/journal.pone.0000665)

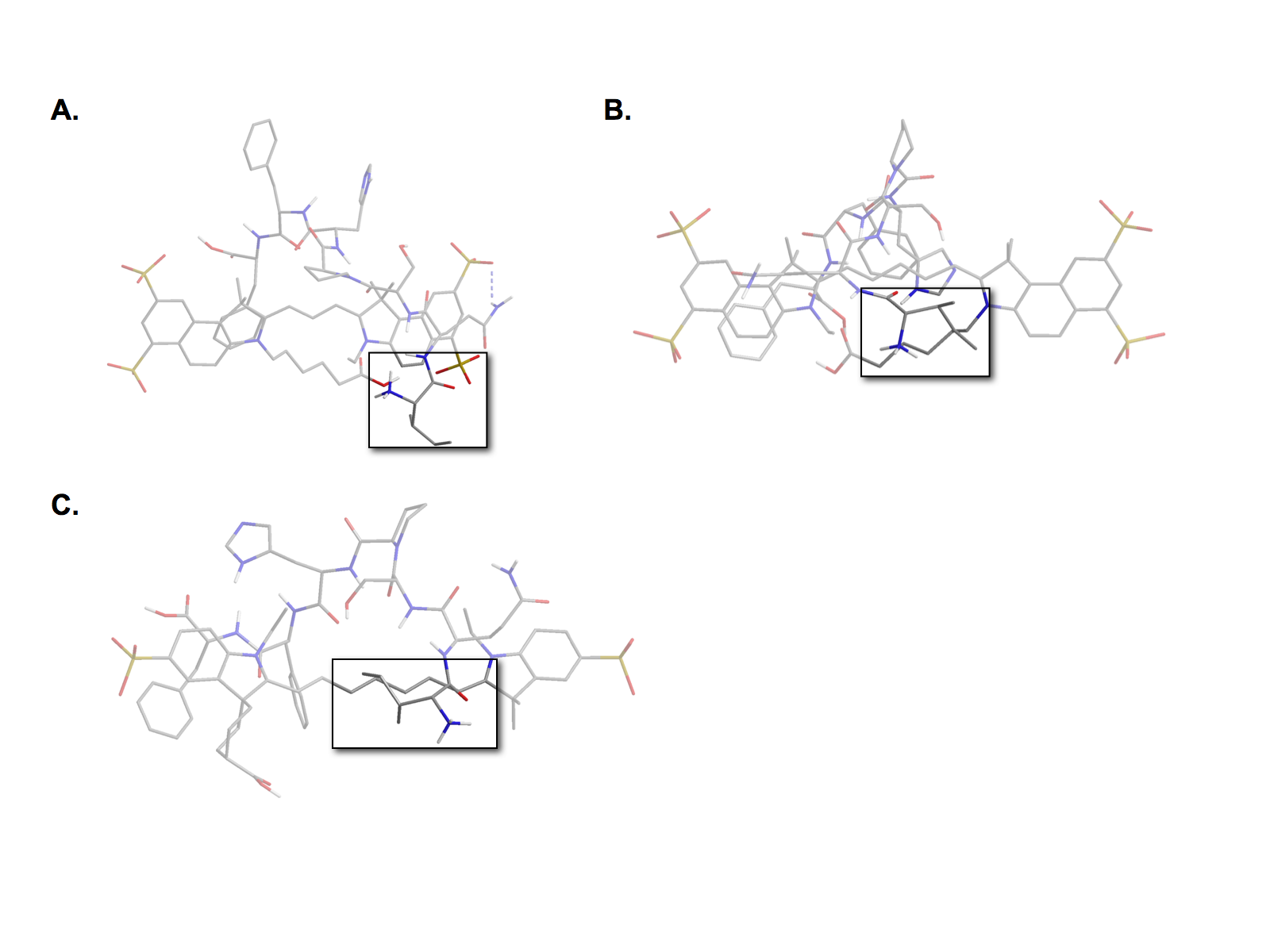

Supplement: Figure S1 — Molecular modeling of IQ-tag peptide binding to representative fluorochromes. A. sub-optimal IQSPHFF docking with Cy 3.5 (3-carbon linker too short). B. sub-optimal IQSPHFF docking with Cy 5.5 (isoleucine-hexanoic acid interference). C. IQSPHFF docking with AF750. (0.21 MB TIF) [file pone.0000665.s001.tif]

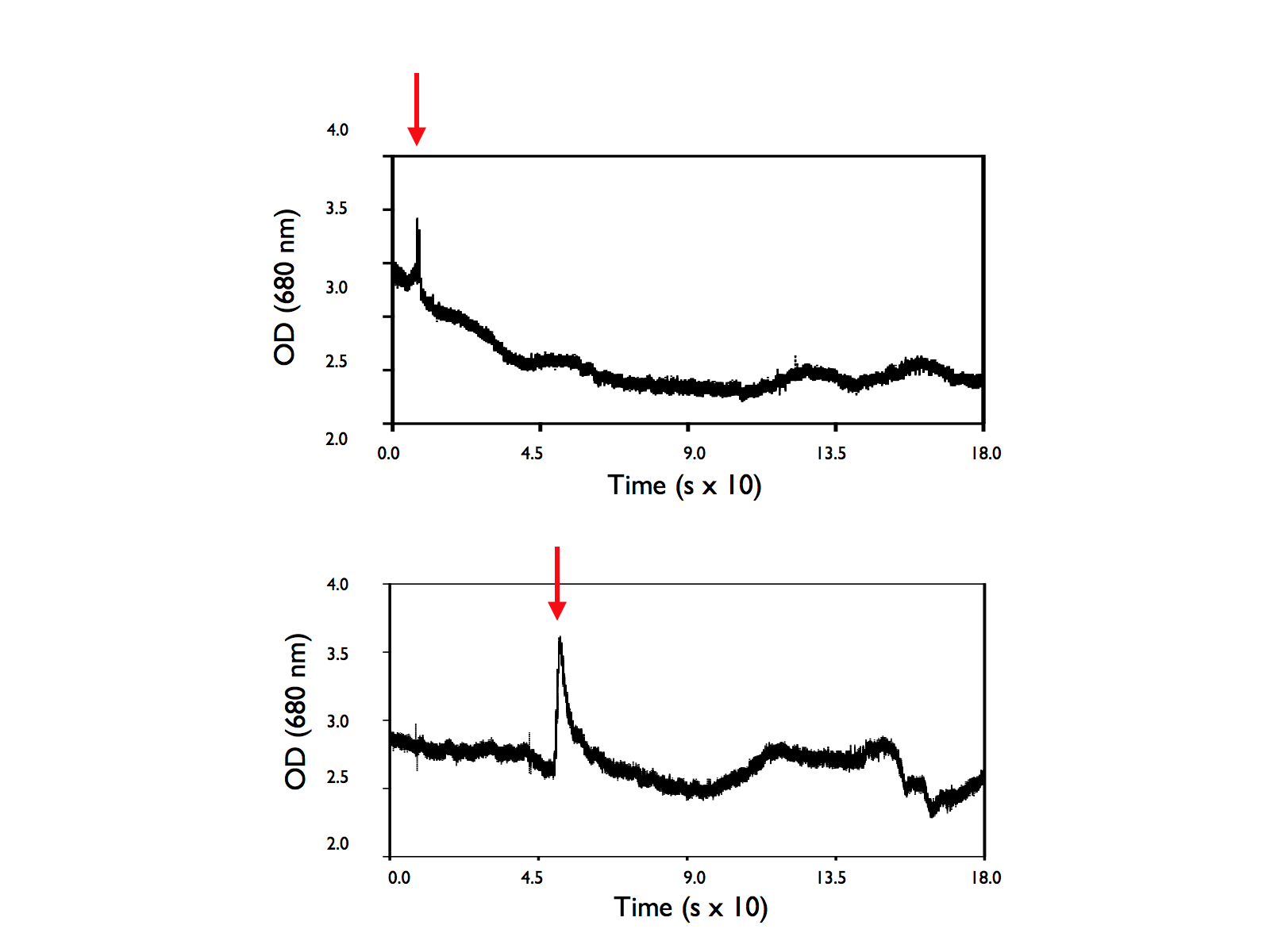

Supplement: Figure S2 — Analysis of IQSPHFF peptide binding to NIRF via HPLC. Free GH680 (upper trace) or GH680 incubated with IQ-tag (lower trace) were analyzed via HPLC with a reverse phase C18 column. Note the shift in retention time for NIRF incubated with peptide indicating the formation of a stable complex. (0.11 MB TIF) [file pone.0000665.s002.tif]
